# From patient notes to prognostication: The revolutionary potential of event‐based foundation models in orthopaedics

**DOI:** 10.1002/ksa.70428

**Published:** 2026-05-20

**Authors:** Alexander M. Bouterse, James A. Pruneski, Felix C. Oettl, Bálint Zsidai, Umile Giuseppe Longo, Thomas Tischer, Michael T. Hirschmann, Kristian Samuelsson

**Affiliations:** ^1^ Department of Orthopaedic Surgery Tripler Army Medical Center Honolulu Hawaii USA; ^2^ Department of Orthopaedic Surgery Balgrist University Hospital, University of Zurich Zurich Switzerland; ^3^ Department of Orthopaedics, Institute of Clinical Sciences, Sahlgrenska Academy University of Gothenburg Gothenburg Sweden; ^4^ Sahlgrenska Sports Medicine Center Göteborg Sweden; ^5^ Department of Orthopedics Skåne University Hospital Malmö/Lund Sweden; ^6^ Department of Orthopaedic and Trauma Surgery Fondazione Policlinico Universitario Campus Bio‐Medico Roma Italy; ^7^ Research Unit of Orthopaedic and Trauma Surgery, Department of Medicine and Surgery Università Campus Bio‐Medico di Roma Roma Italy; ^8^ Department of Orthopaedic Surgery University Medicine Rostock Rostock Germany; ^9^ Department of Orthopaedic and Trauma Surgery Malteser Waldkrankenhaus Erlangen Germany; ^10^ University Department of Orthopedic Surgery and Traumatology Kantonsspital Baselland Bruderholz Switzerland; ^11^ Department of Clinical Research, Research Group Michael T. Hirschmann, Regenerative Medicine and Biomechanics University of Basel Basel Switzerland; ^12^ Department of Orthopaedics Sahlgrenska University Hospital Mölndal Sweden

**Keywords:** artificial intelligence, CoMET, event‐based foundation models, large language models, natural language processing

## Abstract

The integration of artificial intelligence (AI)‐equipped tools into electronic health record (EHR) platforms may drive the evolution of orthopaedic diagnosis and decision‐making, leveraging big data to generate precise and context‐aware insights. With the advent of event‐based foundation models, a critical step forward in predictive modelling is imminent, with upcoming implementations promising prospective generation of patient timelines, anticipated future events and real‐time estimates of risk, even when trained on heterogeneous, multimodal healthcare data. Namely, Epic's Cosmos Medical Event Transformer (CoMET) platforms, a newly introduced family of event‐based models trained on billions of clinical encounters, are capable of learning and accurately predicting temporospatial patterns in medical occurrences at scale, thus enabling a new approach to patient‐specific decision‐making and prognostication. Accordingly, the current manuscript aims to provide a description of these models, including their unique capabilities, potential limitations and future applications to orthopaedic practices.

**Level of Evidence:**

Level V.

**Abbreviations**
AI: artificial intelligence
CoMET: Cosmos Medical Event Transformer
EHR: electronic health record
GPT: generative pre‐trained transformer
LLM: large language model
ML: machine learning
NLP: natural language processing

## INTRODUCTION

A growing interest in the novel applications of artificial intelligence (AI) to clinical and academic pursuits has facilitated the broad incorporation of AI‐powered tools into orthopaedic practices [33, 38, 39, 42, 47, 63]. Specifically, natural language processing (NLP) platforms and large language models (LLMs) have proven capable of rapidly improving the efficiency and adaptability of electronic health records (EHRs) in clinical documentation and data interpretation [1, 4, 15, 16, 18, 46, 49, 56, 63]. As these tools are introduced, the integration of multimodal data analysis is needed to further increase their utility and accuracy. In doing so, decision‐support systems could become capable of interpreting multifaceted medical scenarios and guiding intricate decisions in a manner similar to or potentially surpassing the capabilities of human physicians [3, 22, 26, 51, 53, 60]. As such, recent efforts to develop clinical prediction models have leveraged large quantities of multimodal data from EHR repositories to characterize patient trajectories and predict future events [2, 5]. The resulting platforms, dubbed ‘event‐based foundation models’, incorporate the insights of billions of historical clinical encounters and represent an advancement in the capabilities of adaptive clinical models, providing personalized predictions of future events, reliable prognostication and a framework for decision‐making in orthopaedic practices.

Early examinations of the performance and applicability of these models have underscored a potential to guide new clinical workflows and patient‐specific treatments, demonstrating both their scalability and capacity to outperform established clinic models [57, 62]. Specifically, preliminary testing of Epic's Cosmos Medical Event Transformer (CoMET) models (Epic Systems), a family of event‐based foundation platforms, has illustrated their ability to assimilate large quantities of multimodal EHR data and generate accurate predictions of anticipated medical events based on the temporospatial patterns contained within a patient's unique healthcare history [57]. Collectively, these platforms, amongst other event‐based foundation models, illustrate a notable advancement in medical decision‐making and represent a shift from problem‐specific models toward multifaceted platforms capable of comprehensive risk forecasting. Such models, if deployed with careful validation and governance, may progress the field from siloed scores toward timeline‐aware models that can anticipate complications, streamline workflows and accelerate registry development and utilization. Accordingly, the purpose of this work is to examine the creation, testing and validation of event‐based foundation models and to explore their implications on the future of orthopaedic research and clinical practice.

### Current catalogue of EHR research

To date, much of the research pertaining to the integration of AI‐powered tools within EHR systems has centred around the utilization of NLP and LLM platforms to provide ambient medical documentation and intelligence‐driven patient correspondence. Specifically, NLP software has exhibited a robust ability to provide real‐time interpretation and transcription of physician‐patient interactions into the EHR, resulting in improvements to note‐writing times and overall encounter times as well as provider satisfaction and metrics of clinical burnout [1, 4, 16, 31, 49, 55]. While current research efforts aim to reinforce the efficacy of these tools within orthopaedic practices, future studies may focus on the application of NLP platforms to particularly exacting settings, such as high‐volume arthroplasty centres or sports medicine practices, to investigate their capabilities when strained by significant data input. Similarly, LLM‐supported patient messaging has shown promise through early implementation, with AI‐generated responses to patient inquiries often producing improved accuracy and ratings of empathy when compared to replies authored by physicians [6, 17, 52, 61]. Future studies may serve to further define the role that these platforms could carry in clinical practice, with potential orthopaedic applications including automated responses to non‐urgent post‐operative complaints and AI support during pre‐surgical counselling.

The capabilities of clinical tools embedded within EHR systems have the potential to be greatly enhanced by the introduction of AI in the form of machine learning (ML)‐powered predictive models. Ongoing research concerning the efficacy of these platforms has demonstrated excellent accuracy when stratifying risk for relevant orthopaedic outcomes such as readmission, discharge disposition and periprosthetic joint infection following total joint arthroplasty [7, 8, 12, 13, 25, 36, 41]. While current efforts largely centre around benchmarking such models against established workflows, such as the National Surgical Quality Improvement Program and Risk Assessment and Prediction Tool perioperative risk calculators, future applications may seek to offer dynamically integrated EHR tools that provide continuous estimates of perioperative risk based on patient‐, institution‐ and practice‐specific factors. Furthermore, through interpretation of clinical documentation using NLP software, multimodal data have begun to be incorporated within these models, ushering in improved predictive efficacy and powerful applications of cross‐media data analysis that may transform risk modelling and perioperative decision‐making within orthopaedic practices [34, 35].

A compelling example of multimodal AI architecture is the utilization of EHR transformer models, NLP‐powered platforms that utilize self‐attention mechanisms in order to effectively capture the complex and heterogeneous dependencies and data relationships within EHR structures [10, 20, 32, 37]. Namely, Hi‐BEHRT, a model introduced with the aim of expanding the capabilities of transformer frameworks in the interpretation of long‐sequence EHR data, presents a promising evolution of this concept, with its hierarchical feature extraction and aggregation allowing for improved performance relative to established transformer concepts [30]. Specifically, this model proved capable of yielding consistent improvements in accuracy over benchmark transformer models when tasked with predicting the incidence of select medical conditions using high‐volume, longitudinal and multimodal patient data, including physician documentation, laboratory values, demographic information and procedural results [30]. This model was also capable of interpreting a much greater quantity of EHR entries than standard transformer platforms, further emphasizing its utility in large‐scale EHR analysis and future research [30]. While currently tested on the prediction of well‐defined medical comorbidities, this approach may offer a scalable framework through which diverse data inputs and extended perioperative trajectories could be incorporated into orthopaedic practices.

In light of the ever‐growing volume of research pertaining to AI‐enabled EHR modelling, Ren et al. delivered a unified taxonomy of current and developing platforms, including recent advancements in data‐centric approaches, neural architecture design, learning‐focused strategies, multimodal learning and LLM‐based modelling systems [45]. Though the compendium of available modalities continues to grow, understanding the appropriate applications of these technologies equips orthopaedic practitioners and researchers with an extensible playbook for facilitating controlled implementations of AI tools within clinical practices.

### Event‐based foundation models

Newly introduced event‐based foundation models represent the next evolution of AI‐powered EHR tools (Figure 1). These platforms offer a conceptual departure from traditional EHR foundation models that rely on cross‐sectional representations or aggregate summaries of clinical data, instead modelling healthcare data as a dynamic narrative, through which event‐based models can learn the order and timing of medical occurrences and effectively capture the interplay between clinical events over time [57]. In this framework, every clinic visit, laboratory result, medication administration, imaging report or procedural intervention is treated as a time‐stamped event that can inform models of the temporospatial relationships between these instances. In learning such patterns, these models carry the potential to provide individualized predictions of upcoming medical events and estimates of the probability of select adverse outcomes. For instance, in the preoperative evaluation of a 67‐year‐old female with longstanding diabetes, event‐based foundation models could offer the identification of patient‐ and time‐specific risk factors—escalating haemoglobin A1c values, a recent unplanned emergency department visit, a history of prolonged hospitalization following a previous surgery—and provide estimates of risk for postoperative complications, often with better accuracy than predictions based on isolated variables. Concurrently, probable future events relevant to this patient's perioperative course, such as IV antibiotic administration or a postoperative emergency department visit, could also be produced and serve to assist physicians in anticipating and proactively mitigating complications. When trained on sufficiently large quantities of data, these models could uniquely facilitate a thorough yet generalizable understanding of healthcare trajectories that can be applied to a wide range of downstream tasks without requiring retraining and model fine‐tuning [57].

**Figure 1:**
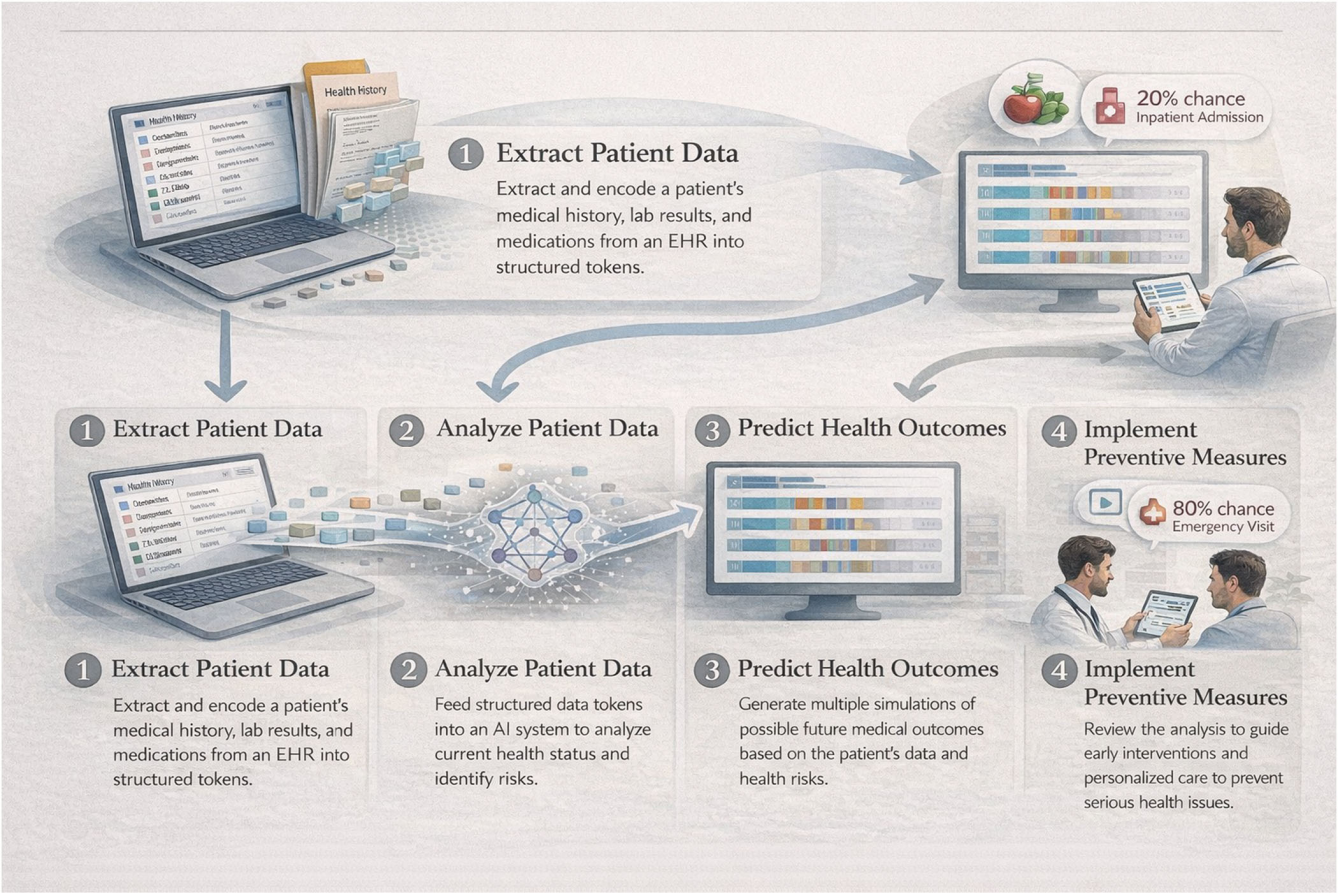
Sequential schematic of how a GPT‐style transformer turns longitudinal EHR data into actionable signals. A patient's time‐ordered events (diagnoses, labs, etc.) are tokenized, along with time‐gap encodings (e.g., ‘+2 days’) and optional static context (age, sex, comorbidities). These tokens are embedded with token + time encodings. Masked self‐attention blocks process the sequence autoregressively, learning patterns in order and timing; during training, the model predicts the next event from past events only. Trained models then produce the predicted next event based on continuously updated data. EHR, electronic health record; GPT, generative pre‐trained transformer.

Several preliminary models aiming to capture these capabilities have been proposed, including PRISM, MOTOR and XTSFormer—EHR‐trained frameworks that can produce context‐specific predictions based on temporal patterns in medical data [29, 44, 48, 59]. These platforms, though limited by training volume and model size, have provided a conceptual framework for the feasibility and broad applicability of event models across a variety of medical outcomes and healthcare settings. Building from this foundation, Foresight, a transformer‐based model trained on the United Kingdom's National Health Service (NHS) data, was introduced as one of the first large‐scale generative EHR models capable of medical event forecasting. This platform achieved precision values between 0.68 and 0.76 in the prediction of anticipated medical events when pretrained on over 810,000 patients gathered from NHS datasets [27]. Currently, efforts are underway to establish Foresight 2, an expanded iteration of this design with the intention of incorporating larger, nationally based datasets in order to improve temporal precision [28].

Epic's newly introduced CoMET platform, a family of event‐based models that are trained on more than 16 billion clinical encounters contained within Epic's Cosmos dataset, presents a promising advancement within this emerging space. Unique to other currently proposed event‐based platforms, CoMET's access to expansive volumes of training data equips its models with comparable or superior predictive efficacy to that of high‐performing task‐specific models when presented with practical clinical objectives [57]. Specifically, in a large‐scale foundational study, these models exhibited consistent discriminative capability across more than 70 downstream outcomes, yielding area under the curve of the receiver operating characteristic curve (AUCROC) values ranging from 0.77 to 0.97 when tasked with prediction of clinical diagnoses, chronic disease progression and operational endpoints such as 30‐day hospital readmission, length of stay and emergency department visits. Importantly, CoMET consistently matched or outperformed conventional supervised prediction models across a range of selected endpoints without requiring task‐specific fine‐tuning, in some instances increasing model performance by as much as 7% relative to supervised baselines. Furthermore, the ability of these models to perform effectively when trained on the often heterogeneous and temporally irregular data contained within the Cosmos dataset illustrates their extensibility within both the context of complex EHR ecosystems and the demands of diverse clinical environments. Though further quantitative assessments of these models and balanced comparisons to existing modalities are warranted prior to adoption, their scale, temporal modelling capabilities and generalizability suggest substantial potential for advancing predictive performance beyond traditional supervised EHR models.

Orthopaedic practices are uniquely positioned to harness the capabilities presented by these platforms, as the evaluation and treatment of musculoskeletal conditions is inherently dependent on temporospatial relationships between disease processes, procedural interventions and comorbid conditions. Namely, the feasibility and appropriateness of orthopaedic interventions are often highly dependent on patient‐ and time‐specific factors that directly influence the likelihood of treatment complications, the course of injury rehabilitation and the utilization of healthcare resources. For instance, consider a patient planned to undergo an outpatient total knee arthroplasty, whose converging time‐sensitive events, such as an elevation in C‐reactive protein levels, a recent intra‐articular steroid injection or preoperative anaemia, signal enhanced vulnerability to postoperative complications. While initially planned for a same‐day discharge, detection of elevated risk by event‐based models could allow for a dynamic assessment of this patient's likelihood of experiencing an avoidable postoperative complication, such as a surgical site infection or unplanned emergency department visit, thereby allowing the treating physician to consider proactive interventions, such as rescheduling this procedure to take place in an inpatient facility. Similarly, if the initially planned procedure is performed in light of these risks, the model's ability to provide early detection of risk indicators—such as postoperative messages describing persistent wound drainage or unexpected escalations in analgesic requirements—could allow it to prompt proactive interventions, such as an in‐person wound evaluation, alteration of the patient's follow‐up schedule or prescription of an antibiotic regimen. This degree of context‐aware analysis exemplifies how event‐based models could transform EHR tools from passive adjuncts to active clinical partners that support adaptive and data‐driven decision‐making in orthopaedic care. While event‐based platforms have yet to be specifically applied to orthopaedic endpoints and much of the evidence supporting their efficacy remains in preprint and has yet to be empirically validated across real‐world healthcare settings, the theoretical benefit held by these modalities carries substantial potential to enhance the effectiveness of musculoskeletal interventions and the efficiency of orthopaedic physicians.

### Scaling laws

An inherent property of modern foundation models is their adherence to a power–law relationship between the scale of their training and computing resources and the resulting model's performance [23, 50]. Simply put, within the framework of these scaling laws, alterations to the model's size, its available volume of training data or its effective computation power, result in a predictable change to its observed error—a relationship that often follows a smooth and quantifiable curve (Figure 2) [23, 50, 62]. Importantly, these relationships are an integral component of the predictive efficacy offered by Epic's CoMET platforms, as their access to billions of historic clinical encounters, in tandem with increasing model size and massive computational power, enables marked improvements to predictive performance. Waxler et al. provided the foundational framework for this principle within CoMET models by measuring the performance of three distinct models of varying size, training volume and computational reserve across 78 downstream clinical tasks, including disease prognosis, diagnostic analysis and outcome forecasting [57]. Across these models, a steady decrease in training losses was observed with upscaling of each sequential model, the largest of which contained one billion data parameters, 1.7 trillion training tokens and 14 billion teraFLOPs of computing power. Consequently, as the scale of these models continues to grow in proportion to the volume of Epic's data repositories and computational resources, their predictive capabilities will continue to expand, providing physicians with the capacity to anticipate, intervene and individualize care with an ever‐increasing level of temporal precision.

**Figure 2:**
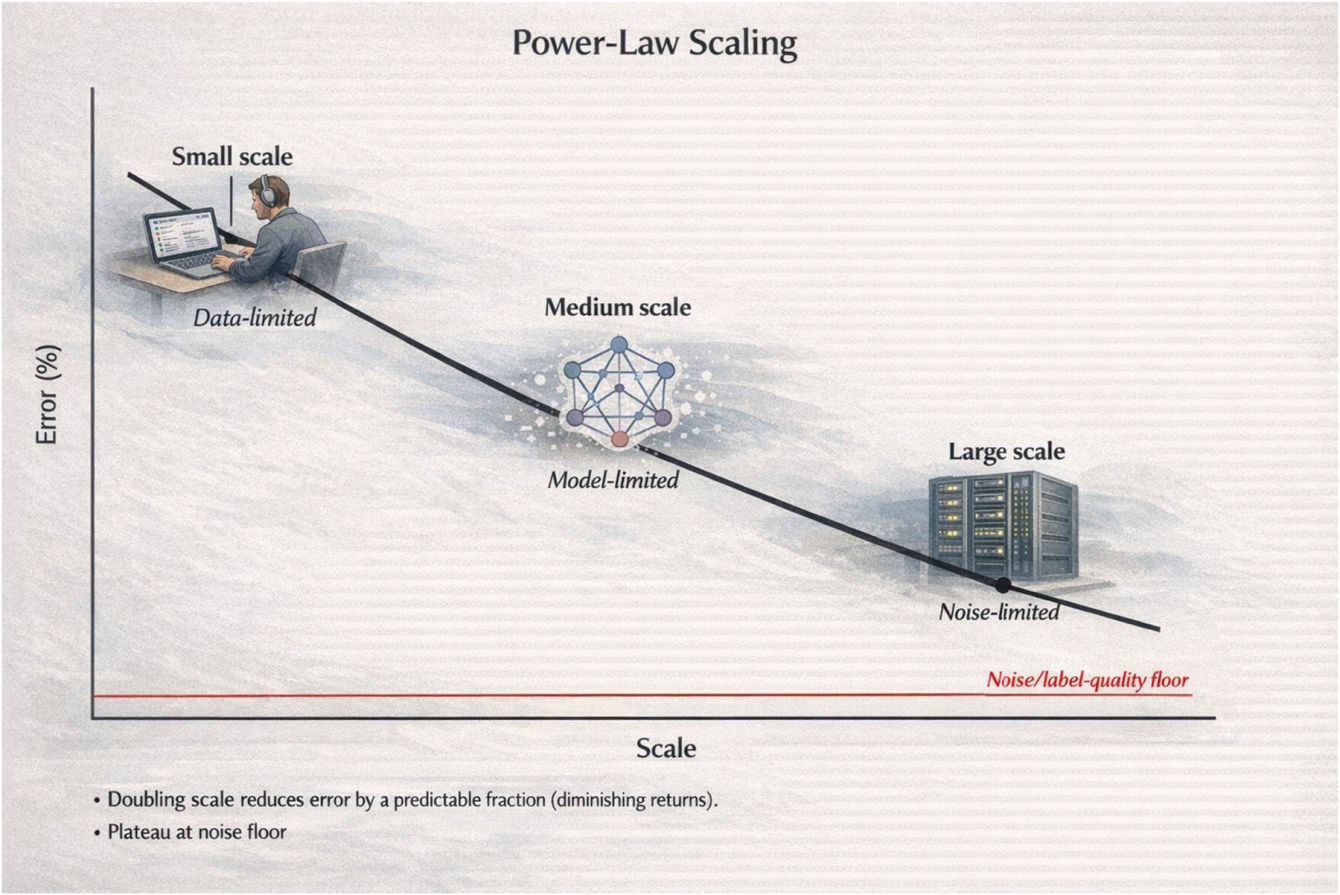
Simplified representation of power‐law scaling. Model error tends to decrease predictably as more training events, bigger models or more computational power is harnessed. However, model error plateaus at a noise floor, which can be affected by label quality and more complete clinical timelines.

Within orthopaedic practices, scaling law principles carry similar importance, as efforts to improve prediction of surgically relevant outcomes using event‐based foundation models are inherently dependent on the availability of both comprehensive data and model scalability. Notably, while CoMET models have performed excellently when tasked with event prediction for common medical diagnoses, such as chronic kidney disease and congestive heart failure, predictions of granular outcomes, such as postoperative readmissions following knee arthroplasty procedures, may be limited by the comparative paucity of available data [57]. As such, scaling laws should be used to guide future efforts to define these outcomes, with additional longitudinal data collection used to supplement deficiencies in training volume, devotion of institutional and infrastructural resources to expand computational budget and architectural refinement to increase the size of the platforms specific to prediction of these endpoints.

While promising for both general and orthopaedic practices alike, scaling law principles are not without limitations. Namely, extrapolation across varying tasks or clinical environments can violate the assumptions incorporated into the model's decision‐making framework, making prediction of patient‐ and practice‐specific outcomes challenging. Furthermore, as these models are trained on inherently heterogeneous EHR, a plateauing of model performance may eventually be observed despite additional scaling due to the noise introduced by inconsistent and poorly structured data inputs (Figure 2). As such, while scaling laws underscore an exciting potential for increasing predictive efficacy through event‐based modelling, physicians and researchers should endeavour to ensure that scaling remains purposeful, interpretable and aligned with both real‐world clinical outcomes and the limitations of these platforms.

### Future directions

Despite the potential carried by event‐based models, their eventual deployment must first be tempered by careful validation and staged integration. Importantly, much of the evidence to support the efficacy of these platforms is limited to singular exploratory studies or preprint sources and has not yet been applied to orthopaedic endpoints, thus limiting their suitability for adoption into clinical settings. If such models are to be implemented into clinical decision‐making frameworks, a structured pathway for clinical evaluation should be implemented, with local retrospective studies assessing accuracy against historical outcome metrics, peer‐reviewed external validation efforts ensuring generalizable performance and silent prospective run‐in periods enabling passive predictions to be generated prior to incorporation into real‐world workflows. For meaningful orthopaedic applications to become feasible, integration with domain‐specific outcome datasets, such as large‐scale arthroplasty registries, and prospective evaluation within perioperative workflows will be required to validate the impact of these platforms on clinical decision making and patient outcomes. Additionally, once sequential integration begins, both in generalized practice and orthopaedic environments, standardized performance thresholds and stop rules should be thoroughly defined to prevent model underperformance from adversely influencing sensitive areas of decision‐making. Such limitations are an arduous yet necessary component of responsible implementation of these powerful tools, as prior endeavours, such as the Epic Sepsis Model—a rapidly integrated risk prediction algorithm that eventually underperformed during external validation—have demonstrated the clinical and logistic consequences that early, unchecked adoption may carry [9, 19, 40, 58]. Furthermore, a clearly established set of reporting guidelines is necessary prior to the widespread application of these models to previously untested outcomes. Namely, the selection and definition of distinct endpoints should be clearly described, while both model calibration methodology and the detailed results of subgroup analyses should be made readily available in order to allow for benchmarking against human‐led workflows.

As steps are taken toward integration, assimilation of EHR platforms with existing orthopaedic databases may bolster the strength of models tasked with examining the outcomes specific to registry‐tracked orthopaedic interventions and patient populations. For instance, the American Academy of Orthopaedic Surgeons has announced cross‐connectivity between its American Joint Replacement Registry and Epic's Community Registries infrastructure—a linkage that will effectively enable real‐time data entry and continuous calibration of the models tasked with prediction of arthroplasty outcomes [24]. As these systems mature, event‐based foundation frameworks may eventually obviate the necessity of outcome registries entirely, with models being fine‐tuned to independently recognize and predict the adverse outcomes recorded within these databases. Accordingly, orthopaedic practices will become increasingly equipped with continuously updated, registry‐grade outcome data that both refines predictive accuracy and ensures that model‐generated recommendations remain aligned with evolving standards of care.

### Ethical considerations

Despite the promise carried by event‐based foundation models, their gradual implementation must be appropriately tempered by an awareness of their capacity to introduce bias and discriminatory practices into decision‐making processes. Specifically, the ability of these platforms to depict the likelihood of adverse medical events potentially creates an ethically fraught environment, in which patients with a greater likelihood of experiencing complications are disproportionately subjected to higher insurance premiums or are not afforded the same treatment options as comparatively younger and healthier patients. Furthermore, as the predictions produced by these models are dependent on the quality of documentation contained within EHR repositories, existing biases within healthcare settings, such as discriminatory recording of perioperative pain ratings in racial and ethnic minorities, may be perpetuated and unintentionally integrated into the decision‐making frameworks produced by event‐based models [21, 43, 54]. As such, orthopaedic physicians seeking to utilize the capabilities of these platforms, must be attuned to the ethical shortcomings inherent to algorithmic decision‐support systems and should ensure that these models are maintained as a supplement to clinical judgement rather than as a determinant of care.

A responsible implementation framework for event‐based foundation models in healthcare should extend beyond aggregate performance metrics. Ongoing governance should include prospective bias auditing, transparent reporting of subgroup‐level performance, monitoring for calibration drift after deployment and clear clinician override pathways when model recommendations are discordant with the clinical context [11, 14]. Together, these safeguards help support fairness, reliability and appropriate human oversight during real‐world adoption.

## CONCLUSION

Event‐based models hold the potential to change the delivery of orthopaedic care, with adaptive, temporally specific predictions of surgically relevant events becoming feasible on both an individual and system‐wide scale. While significant steps toward validation and eventual implementation remain, these models may soon become an increasingly commonplace component of clinical decision‐making and personalized risk assessments. As such, their thoughtful development and responsible deployment by orthopaedic physicians may ultimately serve to shift comprehensive orthopaedic care from a reactive process to an anticipatory, trajectory‐based framework that enhances the precision, efficiency and personalization of our interventions.

## AUTHOR CONTRIBUTIONS

All listed authors have contributed substantially to this work: James A. Pruneski, Alexander M. Bouterse and Felix C. Oettl performed the literature review and primary manuscript preparation. Editing and final manuscript preparation were performed by Bálint Zsidai, Umile Giuseppe Longo, Thomas Tischer, Michael T. Hirschmann and Kristian Samuelsson. All authors read and approved the final manuscript.

## CONFLICT OF INTEREST STATEMENT

Kristian Samuelsson is a member of the Board of Directors in Getinge AB (publ) and medtech advisor to Carl Bennet AB. Thomas Tischer is an Associate Editor for *KSSTA*. Michael T. Hirschmann is the Editor‐in‐Chief of *KSSTA*, and a consultant for Medacta, Symbios and Depuy Synthes.

## ETHICS STATEMENT

The authors have nothing to report.

## Data Availability

The authors have nothing to report.
